# Confidence Levels or Degrees of Sentience?

**DOI:** 10.1007/s41649-022-00230-5

**Published:** 2022-11-10

**Authors:** Walter Veit

**Affiliations:** University of Bristol, Bristol, UK

**Keywords:** Animal sentience, Animal pain, Animal ethics, Pluralism, Degrees of sentience

## Abstract

I applaud recent improvements upon previous guidelines for the assessment of pain in non-human species and the application of their framework towards decapod crustaceans. Rather than constituting a mere intermediate solution between the scientific difficulty of settling questions of animal consciousness and the need for a framework for the purposes of animal welfare legislation, I will argue that the longer lists of criteria for animal sentience should make us realize that animal sentience is a multi-dimensional phenomenon that must be studied with a plethora of methods in order to assess its diversity across the tree of life.

I applaud Crump et al. (2022) for improving upon previous guidelines for the evaluation of whether animals can feel pain and their rigorous review of the literature to assess the capacity of crustaceans (and cephalopod mollusks in their longer report for the Department for Environment, Food and Rural Affairs; see Birch et al. 2021) to feel pain. As the impact of their work on animal welfare legislation has elegantly demonstrated, research on animal sentience has reached a new height in being considered a legitimate subject of scientific investigation. Yet, it is neither in their provision of new animal sentience indicators, nor in the serious consideration of sentience in crustaceans that I see the greatest value of their work. Let me elaborate.

Ever since animal pain and welfare gained serious attention of policy-makers, there has been a tension between the need for official guidelines of animal protection and at the same time the difficulty of studying animal welfare as a state of subjective wellbeing (Browning 2020). Guidelines, such as those by Smith and Boyd (1991) were intended as pragmatic intermediate solutions to the question of which animals should be protected in the absence of a scientific consensus on the neurological basis of consciousness. Many scientists studying animal cognition exhibit something like a double standard for the attributions of sentience when it comes to policy-making and science. As Browning and Birch (2022) have argued, the moral demands of protecting sentient beings force us to rely upon precautionary reasoning in a policy-making context (see also Knutsson and Munthe 2017; Birch 2017; Birch and Browning 2021). So there inevitably appears to be a gap here between scientific criteria for the protection of sentience-contenders and for the purposes of a science of animal sentience, which partially explains why there is such disagreement about the question of how animal consciousness should be studied scientifically (Browning and Veit 2020; Birch et al. 2022). As I shall argue in this commentary, however, the work by Crump et al. (2022) is moving us towards closing this gap.

Recently, Veit and Browning (2021) have argued that in the study of a phenomena as complex as animal welfare, we require a so-called ‘perspectival pluralist’ approach in order to move animal welfare science further (Veit and Browning 2021). Without diving too much into the philosophical intricacies of perspectival pluralism, it can be usefully summarized for as the idea that complex scientific phenomena require a plurality of models, methods, and concepts in order to better understand the target phenomena at hand. This is because scientific knowledge isn’t simply a revelation of objective facts — a Nagelian view from nowhere — but an inter-subjective enterprise in which scientists with different perspectives come to integrate their knowledge to make sense of the world (see also Giere 2006). Rather than treating the search for theories or methods as something like the search for the one true way of understanding the world, perspectivalists emphasize that our scientific knowledge is necessarily limited, only revealing part of the truth, and thus requiring a pluralist attitude to science.

Crump et al. (2022) effectively embody this philosophical attitude towards a science of animal sentience well, since they, instead of arguing for a particular theory of sentience or something like a single litmus tests for its presence, remain strikingly pluralist in their attempts to strike a balance between a wide range of evidential resources to better assess the capacity to feel pain in decapod crustaceans. The term ‘perspectivalist’ is elegant to emphasize that despite the fact that the subjective experiences of other animals are inaccessible to direct observation, we can nevertheless use a plurality of difference sources of evidence to illuminate their feelings; thus shining light on a phenomenon that has been thought to be inaccessible. Indeed, Crump et al. (2022) stay relatively neutral in regards to what consciousness is or what is for, i.e. what it does for the organism. While some of their suggested experimental paradigms rely on traits that have been linked to consciousness, it is precisely in relying on a plurality of indicators that they avoid committing themselves to a single theory of consciousness that may well turn out to be false. In the measurement of a complex phenomenon, we are often best off by developing multiple measurement tools in order to cross-calibrate our measures and better understand the target phenomenon. As Levins (1966) famously argued, truth can be found at the intersection of independent lies or as perspectivalists perhaps better describe it ‘perspectives’, though I admit this term can cause confusion.

Scientific progress has often been conceived as the narrowing of methods; a kind of natural selection process, in which only the best methods remain to provide close to certain evidence. So-called evidence hierarchies and gold standards of evidence are commonly discussed in the social and biomedical sciences, yet are lacking in the study of animal sentience. However, scientists and philosophers of science have been critical of the idea that we should rank different methods and models, maintaining that its is precisely their plurality that makes them an integral feature of science, rather than a bug (Williams 2010; Cartwright and Hardie 2012; Veit 2019). Importantly, this does not mean that we have to deny that no method is any worse than another, but rather that even in the presence of such evidence hierarchies, there remains value in continuing to rely on other measurement methods in addition to what may be perceived as the currently best method, such as in the case of measuring climate change or economic growth. A plurality of methods ensures greater accuracy and confidence, and I very much see this methodological pluralism embodied in the multiple lines of evidence in Crump et al. (2022).

Indeed, it is my hope that the work of Crump et al. (2022) will help us to make this methodological pluralism accepted within the interdisciplinary study of animal sentience. Rather than constituting a necessary intermediate step in a very young science in which the phenomena is still mysterious, yet simultaneously of great ethical and political importance for animal welfare legislation, I believe that these lists will not be replaced with further scientific progress by something like a single litmus test. Indeed, there is hardly any science of a complex phenomenon that has a single test guaranteeing its presence. This is precisely why the medical profession relies on a variety of indicators to establish for instance the presence of cancer. Simplicity may be a virtue we would like to have in frameworks that are meant to guide scientific policy-making, but animal sentience is unlikely to be a simple phenomenon that is either present or not, instead differing in richness across the animal tree of life (Veit 2022; Birch et al. 2020). As the authors nicely demonstrate, the evidence for sentience differs widely even within the crustacean branch of life.

Instead of admitting a large grey-zone in which we have more or less confidence about the presence of animal sentience in different animals, yet struggle to draw the boundaries of animal sentience (see also Veit and Huebner 2020), I urge the authors to take seriously the evolutionary possibility that sentience itself is a gradual matter. Rather than assigning a medium-level of confidence to sentience in penaeid shrimps, we should treat them as case of something we may wish to call quasi-sentience as opposed to human-like sentience. That the capacity to feel is either present or not may have been an attractive idea when sentience was restricted to animals very similar to us, but as we gain confidence that insects, gastropods, cephalopods, and crustaceans have a degree of sentience, it makes less sense to speak of it as a primitive. While policy-making may seem to require a neat boundary, it is not at all inconceivable to have graded levels of protection for different degrees of sentience.

To conclude, future progress will not lead to a litmus test of sentience, but longer more pluralistic lists of evidential criteria and more fine-grained distinctions that will show sentience itself to be ‘pluralistic’: a complex multi-dimensional phenomenon that can come in different varieties across the animal tree of life. And if animal sentience turns out to be such a complex bundle of functional capacities, it will become obvious that sentience itself must be studied with a plurality of methods to understand its diversity as for any other biological trait. Studying the contents and range of an animal’s affective experiences as opposed to the mere presence of feelings will constitute significant scientific progress and enable animal sentience research to directly impact our knowledge of how to improve animal welfare, rather than just tell us which animals deserve protection. Studies of motivational trade-offs will be especially important for the improvement of animal welfare since it is here that we learn about how much an animal prefers to be in one state over another (see also Kirkden and Pajor 2006). It is because of the richness and diversity of affective states that the plurality of methods we use to assess animal sentience is here to stay and only increase further.
